# Emotional labor mediates the associations between self-consciousness and flow in dancers

**DOI:** 10.1038/s41598-023-44730-2

**Published:** 2023-10-13

**Authors:** Xiaohui Liu, Yu Liao, Jiayi Tan, Jifang Duan, Ruixiang Gao

**Affiliations:** 1 School of Music, South China Normal University, Guangzhou, 510006 China; 2 Center for Studies of Psychological Application, Guangdong Key Laboratory of Mental Health and Cognitive Science, School of Psychology, South China Normal University, Guangzhou, 510631 China; 3 School of Chinese Language and Literature, South China Normal University, Guangzhou, 510006 China; 4 Empirical Research Center for Aesthetics and Aesthetic Education, Institute of Advanced Studies in Humanities and Social Sciences, South China Normal University, Guangzhou, 510631 China

**Keywords:** Psychology, Human behaviour

## Abstract

Emotional labor has been a focal point in occupational well-being literature, but studies have long overlooked an important group of emotional laborers: performers. This research represents a pioneering effort to examine dancers’ adoption of emotional labor strategies, their antecedent of self-consciousness, and the outcome of flow experience. We explored these elements both in the traditional setting of stage dancing and in the novel context of online dance classes without on-site spectators during the COVID-19 pandemic. The results revealed that dancers employed all three common emotional labor strategies: surface acting, deep acting, and expression of naturally felt emotions, with deep acting being the most frequent. In the traditional setting, only the expression of naturally felt emotions mediated the positive effect of private self-consciousness and the negative effect of public self-consciousness on flow experience. In contrast, in the online setting, only private self-consciousness impacted flow through the mediation of deep acting and expression of naturally felt emotions. This exploratory study bridges dramaturgy-originated theories of emotional labor with empirical performing arts research, preliminarily advancing knowledge in the relevant fields of dance education, self-presentation, and flow studies.

## Introduction

With an aim to improve sustainability of employee’s occupational development, it is important for organizations to cultivate work environments supportive of employees’ mental health and well-being. Consequently, emotions in work have received a burgeoning rise of research attention in the field of organizational and management psychology. Emotional labor, first proposed by sociologist Arlie Russell Hochschild in 1983, is the idea of managing emotions to create a publicly appropriate display according to the occupational demands^1^. It is regarded as the third kind of work role, parallel to physical labor and intellectual labor, that is most prototypical in front-line jobs which require “service with a smile”^2^. As the organizational sciences literature has gradually recognized the value of understanding emotions at work^3^, the past three decades has seen an unprecedented growth in the focal area of emotional labor research^4^. While emotional labor examination has been done on a variety of service organizations or workplaces, such as hotels, banks, hospitals, airports, stores, call centers, etc.^5^, the theory, in fact, originated from a “dramaturgical” perspective comparing service encounters to theatrical performances^1^. However, despite this dramaturgical origin, there has been a significant gap in empirically examining emotional labor processes among professional performers and actors.

Dancers represent an insightful, albeit complex, performing arts group to investigate emotional labor theories given the emotion-laden and diverse nature of their work. Expressing appropriate emotions through their dancing is a crucial occupational requirement. However, no research has specifically explored dancers use of emotional labor strategies or linked them to indicators of dancers’ performance and well-being. Testing emotional labor theories in dance contexts is particularly insightful, as it not only provides novel evidence to strengthen and expand the framework beyond traditional service industry settings but also brings the investigation back to the setting on which the concepts were originally based.

In the current study, we aimed to address this research gap by investigating the emotional labor strategies utilized by dancers and relating these strategies to dancers’ self-consciousness as the antecedent and their experience of flow as the subsequent well-being outcome. Self-consciousness in this context refers to an acute awareness of oneself, influencing how individuals perceive their role and interactions with the spectators during a performance. Flow, on the other hand, represents a state of optimal experience characterized by focused attention, loss of self-consciousness, and enhanced intrinsic motivation, which is particularly relevant for performance-based occupations like dancing. Exploring these uncharted relationships for dancers bridges dramaturgical theories of emotional labor and empirical performing arts research, advancing conceptual knowledge of emotional labor in an underexamined yet highly relevant occupational context and offering practical implications for facilitating well-being among this understudied group of performers.

### Emotional labor strategies of dancers

Interestingly, although emotional labor of actors has rarely been empirically studied, Hochschild’s definition of emotional labor^1^ actually stemmed from the “dramaturgical” perspective of server-customer interactions, which compared service employees who carried out emotional labor at the workplace for gratification of the customers to actors “employing expressive devices” at the stage to entertain the audience^6^ (p. 430). There are two major ways for such employment of expressive devices. According to the Stanislavski method of acting^7^, drama actors are encouraged to truly feel the emotions of the characters that they portrayed at any given moment. In contrast, other viewpoints, such as those of Brecht and Meyerhold, “considered an emotionally detached actor more capable of arousing maximum emotional effects in the spectator”^8^ (p. 132). The school of Chinese Mei Lanfang also held that actors should play the characters in a stylized and fixed way without the need of inhabiting authentic emotions while on stage^9^. Similarly, there are two possible strategies for employees to conduct emotional labor. To achieve appropriate outward manifestation of emotions for their occupational goals, employees can either pursue to modify the inner feelings through deep acting (DA), or put efforts into just projecting certain facial expressions or forms of body language through surface acting (SA) without actually experiencing the emotions displayed^1^. Both of these types of emotion regulation processes to reach congruence between emotional requirements from the jobs and the corresponding emotional performance of individual employees have been widely identified in nearly all trades and professions that involve human interaction^10^.

Ashforth and Humphrey argued that focusing on DA and SA only might ignore the possibility that individuals at work were sometime able to spontaneously experience and display the appropriate emotions though they might still have to put forth some conscious efforts to ensure that these feelings and displays coincide with the organization’s expectations^11^. Therefore, Ashforth and Humphrey considered the expression of naturally felt emotions (ENFE) to be a third kind of emotional labor strategies^11^, and this tripartite dimensionality of emotional labor strategy was later confirmed by Diefendorff et al.’s empirical research^12^. For acting itself, the genuine way of ENFE also echoes Brook and Grotowski’s approach of self-expression, but its ultimate objective is identical to those of the other two main streams (Stanislavski versus Brecht) mentioned above, which is that the actors should present his most inner self on stage^8^. Nevertheless, these theatrical theories with respect to actors’ expression of emotions have been developed in the 60s and 70s, yet few empirical evidence has been provided for the debate. Given that drama actors’ use of emotional labor strategies might be a fixed result of their training from a certain acting school, the major purpose of this present paper is to examined emotional labor strategies of a similar group, dance performers, whose area does not have such strong theoretical schools in terms of emotional expression. And the first research question of our study is whether the tripartite dimensionality of emotional labor strategy holds up for dancers, i.e., whether SA, DA, and ENFE are three distinct methods for dancers to display desired emotions.

### Self-consciousness as the antecedent of dancers’ emotional labor

In emotional labor research, Grandey’s integrative conceptual framework is a classic model that is often applied and tested in quantitative studies, which posits that individual difference antecedents and individuals’ well-being outcomes are generally mediated by the emotional labor strategies they adopted^10^. For individual difference antecedents, age, sex, personality traits, work motives, and emotional labor abilities have shown associations with emotional labor strategies^13^. In the present study, we aimed to focus on an individual characteristic more relevant to the self-presentation of dancers—self-consciousness.

According to the self-presentation theory, when one hopes to leave a good impression on others, they are presenting themselves^14–16^. However, although everyone is constantly presenting themselves, there are significant individual differences in the degree of attention they pay to their public image^17^. Self-consciousness is the “consistent tendency of persons to direct attention inward or outward” (p. 552) and is therefore conceived as having two major components, private self-consciousness (PrSC) and public self-consciousness (PuSC)^18^. People with a higher level of PuSC are not only concerned more about their physical appearance and overt behaviors but also more sensitive to others’ gaze, evaluations, and reactions towards their own appearance and behaviors, whereas those with a higher level of PrSC tend to think and reflect more about internal states and are less likely to yield to social pressure and alter their acts according to others’ thoughts and feelings^19–22^. Accordingly, we can develop the following hypotheses linking self-consciousness to the use of emotional labor strategies.

Publicly self-conscious individuals are attuned to role expectations, sensitive to social cues, and accustomed to using these cues to regulate their behaviors^23–25^. This heightened awareness of constant evaluation may prompt them to prioritize the regulation of observable expressions, potentially compromising inner emotional alignment. Consequently, this inclination can foster a reliance on SA, where genuine feelings are suppressed to align with external expectations^26,27^, leading to a corresponding decrease in the utilization of DA and ENFE. This is because aligning internal emotions with external expectations becomes particularly challenging when the emphasis is on managing observable expressions. These dynamics are likely to be especially pronounced for dancers, who frequently perform under intense public scrutiny and pressure^28,29^. Based on this, we hypothesize that dancers’ PuSC is positively associated with their use of SA and negatively correlated with both DA and ENFE.

#### H1:

PuSC is negatively associated with ENFE.

#### H2:

PuSC is negatively associated with DA.

#### H3:

PuSC is positively associated with SA.

At the same time, individuals with high Private Self-Consciousness (PrSC) aim to enhance their performance through continuous monitoring and evaluation of their behaviors^18^. However, in contrast to those with high PuSC, they are more focused on ensuring the congruence between their public presentation and their own beliefs and attitudes, rather than adapting to the expectations of others^30,31^. This steadfastness in maintaining internal alignment across different situations suggests a potential positive correlation with DA and ENFE, as both involve authenticity and consistency in emotional expression. Correspondingly, individuals with high PrSC are less likely to engage in SA, an insincere and tactical self-presentation strategy^32^. Thus, we hypothesize that dancers with high PrSC are likely to exhibit a positive association with both DA and ENFE, and a negative association with SA.

#### H4:

PrSC is positively associated with ENFE.

#### H5:

PrSC is positively associated with DA.

#### H6:

PrSC is negatively associated with SA.

### Flow experience as the outcome of dancers’ emotional labor

In emotional labor research, employee well-being (e.g., job satisfaction, job burnout) is the most commonly discussed outcome of emotional labor strategy adoption, such that SA is the most problematic, that DA is less but still detrimental, and that ENFE is the most beneficial^4,33,34^. The dissonance or inauthenticity mechanism and the resource gains/losses mechanism are the two significant theoretical approaches to make sense of these findings (for a review, see^13^). However, as researchers have pointed out that since employee’s emotional labor is dynamic and varies across time and tasks^35–37^, employee well-being outcomes that could only be manifested in the long run should be better revealed by longitudinal analyses^38–40^. Thus, in this current cross-sectional study, we decided to examine a well-being outcome which is highly momentary and instantaneous and is also of vital relation with dancers’ performance—flow experience, an optimal positive psychological state characterized by deep engagement, absorption, and enjoyment^41^. This concept, introduced by Csikszentmihalyi in 1975^42^, has been the focus of a large body of empirical work in positive psychology spanning over four decades^43^ because of its prominent function of promoting people’s well-being^44^.

The literature claims three essential conditions that lead to the experience of flow: a defined set of goals, a balance between challenges and skills, and the availability of immediate and clear feedback^45^. Certain activities such as dancing are more conducive to flow as they create a challenging and goal-directed setting for the participants and provide more feasible feedback structures that the participants can utilize to know about their progress in developing new skills^46^. However, existing quantitative research on flow experience among dancers is very limited^47^, so our study probing into the relationships between dancers’ flow and use of emotional labor strategies is meaningful.

Both the dissonance or inauthenticity mechanism and the resource gains/losses mechanism can shed light on the possible consequences of emotional labor strategy adoption on flow experience—DA and ENFE might contribute to flow, whereas SA could produce negative or null effects. According to the cognitive dissonance theory^48^, SA requires one to be incongruent with the self^1^, and such dissonance between the mind and the body violates the fundamental and inherent nature of flow. However, since DA “involves deceiving oneself as much as deceiving others”^1^ (p. 33), its consistency at least lays the foundation for the occurrence of flow despite some costs. As long as one is enjoying and devoted to the process of such “deceiving”, they might be able to experience flow therein. And because the authenticity of ENFE enables one to stay true to himself or herself, it is most likely to result in flow experience.

From a different perspective of the resource conservation theory^49^, emotional labor is the process that “workers invest their personal and social resources to help them cope with the service encounter and in anticipation of being able to recoup their investment as a result of the service encounter”^50^ (p. 64). However, except for ENFE that scarcely depletes psychological resources, both DA and SA have different degrees of emotional energy consumption, but which one is more of a drain remains disputed at present^51,52^. In addition, there is also a difference in the social resource gains brought by SA, DA, and ENFE to compensate the energy loss. Deep actors actively reappraise the negative events and try to arouse positive feelings inside, which itself is a process of deriving mental resources^53^. Moreover, displaying genuine positive emotions such as enthusiasm, care, and sympathy from the bottom of the heart is more likely to elicit positive interpersonal feedback from the targets of emotional labor, which not only functions as an important source of social support but also in turn reinforces the employee’s motives and efficacy to continue DA and ENFE^54–59^. Such a positive loop provides a favorable condition for the rise of flow. By contrast, SA signals a lack of skills to successfully manage the challenging and demanding emotion regulation process at work and often results in negative feedback such as dissatisfaction and anger from the targets of emotional labor^54,60^, such that there is a net loss in emotional resources for surface actors over time. All in all, the hypothesized relationships between emotional labor strategies and flow are summed as follows.

#### H7:

ENFE is positively associated with flow experience.

#### H8:

DA is positively associated with flow experience.

#### H9:

SA is negatively associated with flow experience.

### The relationships between self-consciousness and flow experience

While the above nine hypotheses have already formed a mediating model in line with Grandey’s conceptual framework^10^, the antecedent (self-consciousness) might have a direct impact on the outcome (flow experience) without the mediation of emotional labor strategy adoption. As research has demonstrated that individuals high on PuSC are more likely to experience negative emotions such as embarrassment, anxiety, and fear in response to the perceptions of others^61,62^, it can be inferred that PuSC negatively correlates with flow experience. Meanwhile, people with high PrSC tend to have a preoccupation with internal states or an open receptivity to them, and this disposition is believed to overlap with another similar psychological construct of “mindfulness”, a desirable quality of consciousness, awareness, or attention of what is taking place in the present, which is proved facilitate the maintenance and enhancement of well-being^63,64^. Consequently, PrSC is expected to be positively associated with flow experience.

#### H10:

PuSC is negatively associated with flow experience.

#### H11:

PrSC is positively associated with flow experience.

**Figure 1:**
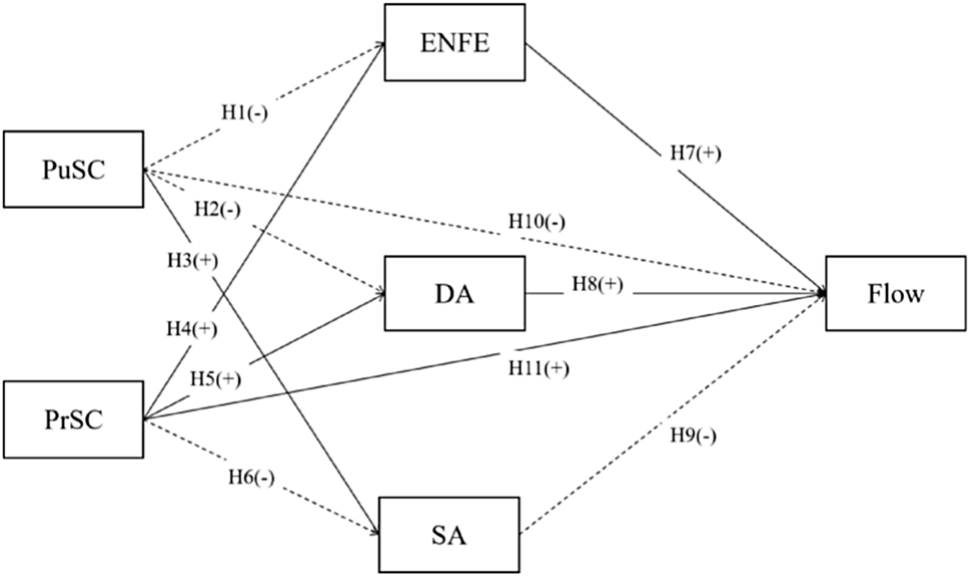
The hypothesized mediation model.

Figure 1 summarizes all 11 assumptions in a mediating model in the light of Grandey’s framework^10^, and whether this model can be successfully established remains the second important question that our current research intended to resolve.

### The present study

To our best knowledge, this is the first empirical study on the emotional labor strategies of dancers as well as its antecedent and outcome. As mentioned above, there are two main research questions in our study. The first one is to verify whether the three-dimensional structure of emotional labor strategies (ENFE, DA, and SA) is tenable for dance performers. As is advised by Nunnally and Bernstein^65^, this question relates to the construct validity of the emotional labor strategy scale for dancers and can be assessed by exploratory factor analysis (EFA) and confirmatory factor analysis (CFA). The second question is to test the eleven-hypothesis mediation model, which is often conducted through structural equation modeling or the improved causal steps approach according to Wen and Liu^66^. Answering these two questions would add to the literature regarding the application of emotional labor theories (e.g., the Grandey’s classic model, research conservation theory) in the field of dance research and would bring practical implications concerning how to enhance the well-being of dance performers^67^.

What makes the present study even more interesting is that two samples of dance major college students were recruited during their daily dance training and show rehearsal to complete the same sets of questionnaire that consists of three scales measuring self-consciousness, emotional labor strategies, and flow experience, respectively. While the first sample of students were dancing together at the stage as usual, the second sample were attending the online dance class and dancing alone to the camera as a result of the closure of colleges during the COVID-19 pandemic, which provided us with a perfect opportunity to explore the difference in dancers’ emotional labor strategy employment without spectators on site. Examining dancers’ emotional labor in different situations would better enrich our knowledge about its nature, as Grandey has pointed out that emotional labor is actually an integrated process of emotional requirements (environmental stimulus), emotion regulation (intrapsychic response), and emotion performance (interpersonal behavior)^13^.

## Methods

### Participants

Two samples of dance major college students were recruited as the participants to complete the three scales of SC, emotional labor strategies, and flow. These two samples received dance training and prepare for dance shows on a daily basis and can be viewed as identical to each other in terms of the level of dancing skills.

Sample 1 (*N* = 184, among them 175 females, *M*_age_ = 18.636, *SD*_age_ = 0.913) completed the questionnaire on a break of regular dance training in the physical offline environment. However, Sample 2 (*N* = 77, among them 74 females, *M*_age_ = 19.026, *SD*_age_ = 1.181) completed the questionnaire during the pandemic of COVID-19, when they were required to continue daily dance training in front of the camera at home and even rehearse remote online performances in such a virtual manner that dancers coordinated with each other through the Zoom meeting. The sizes of the two samples were relatively small but acceptable owning to the rareness of dancer participants.

As these two samples were analyzed separately due to distinct dancing environments and manners.

### Measures

#### Self-consciousness

The Chinese version of Self-Consciousness Scale is a 17-item instrument intended to measure PuSC (7 items, e.g., “I’m always trying to figure myself out”) and PrSC (10 items, e.g., “I’m concerned about the way I present myself”)^68^. The items were presented on a five-point scale five-point Likert-type scale from 1 (*Agree Strongly*) to 5 (*Disagree Strongly*), and each sub-scale was scored by the mean value of corresponding items. The McDonald’s ω values for PuSC and PrSC, respectively, were 0.845 and 0.795 in Sample 1, and were 0.812 and 0.753 in Sample 2, indicating good internal stability of the two sub-scales.

#### Emotional labor strategies

The scale of emotional labor strategies for dancers was adopted and modified from Yin’s Teacher Emotional Labour Strategy Scale^69^, which consisting of three dimensions of SA, DA, and ENFE. In the revision, we mainly changed the wording regarding the emotional labor target from students to spectators to make the items suitable for the dance context. For Sample 1, there were five items, four items, and three items for SA, DA, and ENFE, respectively. For Sample 2, not only the questionnaire guideline was set to direct the participants to the online dancing context, but also three items specifically indicating the online dancing context was added to each sub-scale respectively, so there were six items for SA, five for DA, and four for ENFE. The scale is presented in the appendix. All the items were scored on a five-point Likert-style scale from 1 (*Agree Strongly*) to 5 (*Disagree Strongly*), and the mean values of corresponding items were calculated for the three sub-scores, respectively. The reliability and the construct validity of the scale are assessed and reported below.

#### Flow experience

The Chinese version of Flow State Scale consisted of 33 items for nine aspects: balance (four items), merging (three items), goals (three items), feedback (four items), concentration (four items), control (three items), consciousness (four items), time (four items), and autotelic (four items)^70^. The scale guideline introduced the first sample of dancers to recalling and reporting their feelings engaged in the regular dancing activities, but for the second sample, pointed to the online dancing activities during the pandemic. For each participant, averaging all 33 items, scored on a five-point scale from 1 (*Agree Strongly*) to 5 (*Disagree Strongly*), to form a final point indicating their depth of entering the flow state while dancing. The McDonald’s ω was 0.935 in Sample 1 and was 0.909 in Sample 2.

#### Statistical analyses

The two sample were analyzed separately. For each, factor analyses were conducted first to answer the first research question of the study—whether the three-dimensional structure of emotional labor strategies (ENFE, DA, and SA) was tenable for dancers. Then, as the sample size was relatively small and might be unfitted for structural equation modeling, the improved causal steps approach^66^ was employed to examine the 11-hypothesis mediation model in Fig. 1 so as to address the second question.

### Ethical approval

The study was conducted in accordance with the Declaration of Helsinki, and approved by the Human Research Ethics Committee for Non-Clinical Faculties in School of Psychology, South China Normal University (protocol code: SCNU-PSY-2022-180; date of approval: 15 June 2022). Informed consent was obtained from all participants involved in the investigation.

## Results

### Reliability and construct validity assessment for the scale of emotional labor strategies of dancers

In Tables 1 and 2, we present the results of EFA, CFA, and McDonald’s ω calculation for the emotional labor strategy scale in Sample 1 and Sample 2, respectively. It can be seen that for Sample 1, both internal reliability and construct validity reached good conditions. However, for Sample 2, while the results for EFA and McDonald’s ω calculation were satisfactory, the approximate fit indices in CFA were not so desirable perhaps because of the relatively small but passable sample size (KMO = 0.747 < 0.8, *df* = 105, *p* < 0.001), but the model could still be considered acceptable as it passed the most direct and important test of model fit (*χ*^2^/*df* < 3, *p* < 0.001) suggested by Barrett^71^. These results indicated that the tripartite dimensionality of emotional labor strategies (SA, DA, and ENFE) held up for dancers. Thus, the first research question is addressed.

**Table 1:** Reliability and construct validity assessment for the scale of emotional labor strategies of dancers in Sample 1.

Assessment	Item	Factor loadings			Communality
		Factor 1	Factor 2	Factor 3	
Exploratory factor analysis	SA1	0.785			0.655
	SA2	0.735			0.595
	SA3	0.739			0.713
	SA4	0.787			0.649
	SA5	0.724			0.614
	DA1		0.846		0.778
	DA2		0.858		0.810
	DA3		0.753		0.655
	DA4		0.834		0.756
	ENFE1			0.832	0.711
	ENFE2			0.773	0.664
	ENFE3			0.723	0.762
Internal consistency	McDonald’s ω	0.877 > 0.7	0.809 > 0.7	0.875 > 0.7	–
Confirmatory factor analysis	*χ*^2^	76.624			
	*df*	51			
	*χ*^2^/*df*	1.502 < 3			
	*p*	0.012 < 0.05			
	CFI	0.975 > 0.95			
	TLI	0.967 > 0.95			
	RMSEA	0.052 < 0.08			
	SRMR	0.057 < 0.08

**Table 2:** Reliability and construct validity assessment for the scale of emotional labor strategies of dancers in Sample 2. ^*^Items were only used in Sample 2.

Assessment	Item	Factor loadings			Communality
		Factor 1	Factor 2	Factor 3	
Exploratory factor analysis	SA1	0.801			0.662
	SA2	0.572			0.420
	SA3	0.745			0.585
	SA4	0.805			0.681
	SA5	0.550			0.497
	SA6*	0.806			0.690
	DA1		0.816		0.724
	DA2		0.877		0.779
	DA3		0.849		0.745
	DA4		0.819		0.681
	DA5*		0.857		0.736
	ENFE1			0.827	0.693
	ENFE2			0.840	0.723
	ENFE3			0.726	0.716
	ENFE4*			0.837	0.724
Internal consistency	McDonald’s ω	0.872 > 0.7	0.933 > 0.7	0.900 > 0.7	–
Confirmatory factor analysis	*χ*^2^	165.942			
	*df*	87			
	*χ*^2^/*df*	1.907 < 3			
	*p*	0.000 < 0.001			
	CFI	0.867 < 0.9			
	TLI	0.840 < 0.9			
	RMSEA	0.109 > 0.1			
	SRMR	0.102 > 0.1			

### Preliminary analysis

Descriptive statistics and Pearson correlation for the studies variables in Sample 1 and Sample 2 are depicted in Tables 3 and 4, respectively. It showed that dancers in both samples tended to implement DA while dancing.

**Table 3:** Descriptive statistics and Pearson correlation for the studied variables in Sample 1.

	*M*	*SD*	PuSC	PrSC	SA	DA	ENFE	Flow
PuSC	3.634	0.538	1					
PrSC	3.495	0.424	0.531***	1				
SA	3.364	0.743	0.287***	0.174**	1			
DA	3.878	0.650	0.195**	0.213**	0.359***	1		
ENFE	3.395	0.739	− 0.092	0.113	0.027	0.459***	1	
Flow	3.305	0.442	0.178*	0.439***	0.187*	0.426***	0.441***	1

*p < 0.05, **p < 0.01, ***p < 0.001.

**Table 4:** Descriptive statistics and Pearson correlation for the studied variables in Sample 2.

	*M*	*SD*	PuSC	PrSC	SA	DA	ENFE	Flow
PuSC	3.758	0.477	1					
PrSC	3.635	0.53	0.426***	1				
SA	3.346	0.625	0.055	− 0.096	1			
DA	3.932	0.566	0.138	0.448***	0.045	1		
ENFE	3.308	0.691	0.197	0.373***	− 0.262*	0.247*	1	
Flow	3.296	0.352	0.084	0.389***	− 0.171	0.480***	0.56***	1

*p < 0.05, **p < 0.01, ***p < 0.001.

Also, Harman’s one-way test was performed to examine the common method biases in the two samples, respectively. Putting all items into EFA, before rotation, the first factor (the one with the largest eigenvalue) variation was 21.934% for Sample 1 and 22.437% for Sample 2, both less than 40%, indicating the common method bias had no serious effect.

### Test of the hypothesized mediation model

**Figure 2:**
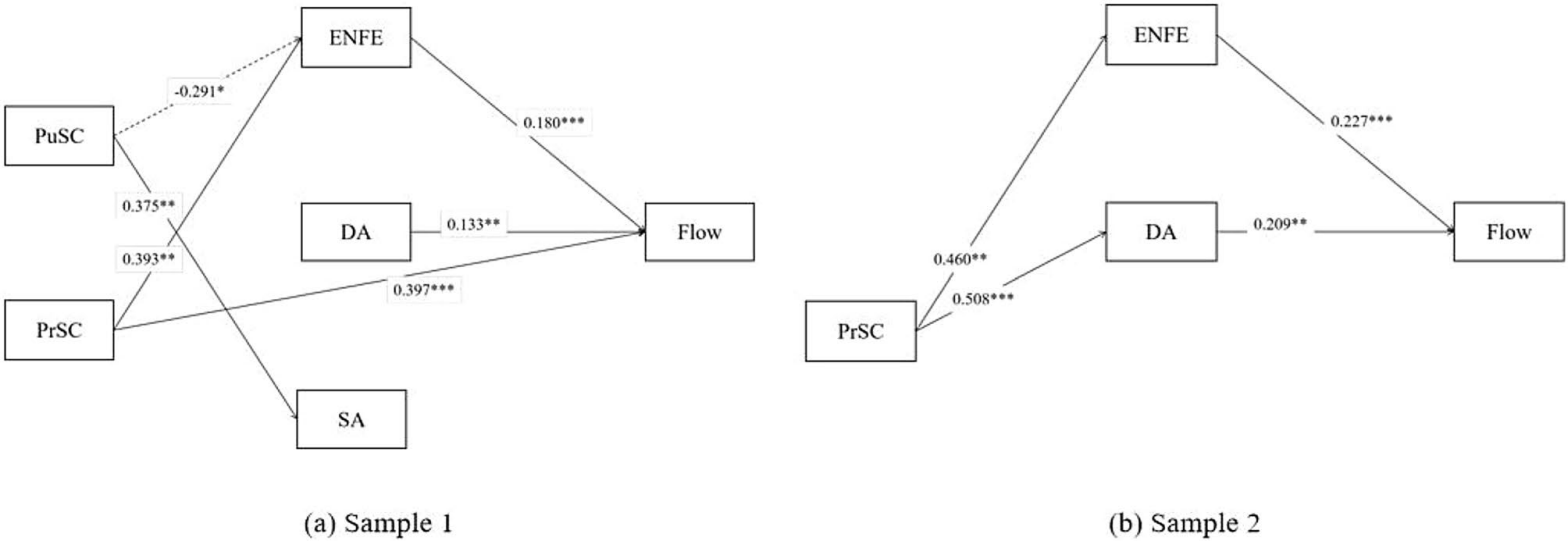
Verification of the research model in Sample 1 (**a**) and Sample 2 (**b**). Non-significant pathways and variables were removed from the model for clarification.

The improved causal steps approach developed by Wen and Liu^66^ was applied to examine the 11-hypothesis mediation model in Fig. 1 so as to answer the second research question of this study. For Sample 1, as is revealed in Table 5 and Fig. 2a, there were one complete mediation path “PuSC → ENFE → Flow” and one partial mediation path “PrSC → ENFE → Flow”, which suggested that in the condition of dancing to spectators, while PuSC of dancers negatively related to their flow experience, PrSC positively related to flow, and that both impacts were mediated by ENFE only. For Sample 2, as is revealed in Table 6 and Fig. 2b, there were two complete mediation paths “PrSC → ENFE → Flow” and “PrSC → DA → Flow”, which indicated that in the condition of dancing without spectators on site, only PrSC related to flow experience in a positive way, and that this impact was mediated by both ENFE and DA. The disfunction of PuSC during online dancing might be accounted for the fact that without facing the spectators, dancers did not need to pay much attention to their outer public images and therefore could focus more on the inner self, which further led to the functional mediating role of DA.

**Table 5:** Test of the mediating effects for Sample 1.

	Regression 1	Regression 2	Regression 3	Regression 4	Regression 5
	Flow	ENFE	DA	SA	Flow
Independent variables					
PuSC	− 0.064	− 0.291*	0.138	0.375**	− 0.043
PrSC	0.500***	0.393**	0.233	0.053	0.397***
Mediating variables					
ENFE					0.180***
DA					0.133**
SA					0.034
R^2^	0.197	0.045	0.055	0.083	0.385
*F*	22.206***	4.253*	5.232**	8.195***	22.293***

*p < 0.05, **p < 0.01, ***p < 0.001.

**Table 6:** Test of the mediating effects for Sample 2.

	Regression 1	Regression 2	Regression 3	Regression 4	Regression 5
	Flow	ENFE	DA	SA	Flow
Independent variables					
PuSC	− 0.074	0.067	− 0.076	0.153	− 0.069
PrSC	0.287***	0.460**	0.508***	− 0.172	0.071
Mediating variables					
ENFE					0.227***
DA					0.209**
SA					− 0.030
R^2^	0.160	0.141	0.204	0.020	0.456
*F*	7.041**	6.054**	9.488***	0.773	11.910***

*p < 0.05, **p < 0.01, ***p < 0.001.

## Discussions

### Theoretical contributions

As an exploratory endeavor into the relatively understudied domain of emotional labor among performers, this study provides preliminary insights that could potentially inform and enrich the existing literature. Firstly, it represents one of the initial attempts to empirically examine the emotional labor strategies of dancers, identifying the presence and frequency of SA, DA, and ENFE adoption in the dancing process, with DA being observed as the most prevalent. Since the majority of participants are on the path to becoming professional dancers, this observation indicates the desirability of cultivating genuine inner emotions that align with the expected roles in dance performances, especially from the Stanislavski perspective^7^. Given these findings are consistent with prior emotional labor literature in other occupations, this study serves as an important, yet previously missing, empirical link bridging the gap between the field of acting studies and vocational research, and underscoring the dramaturgical origins of emotional labor theory^1,6^.

Secondly, this study also attempts to explore the uncharted role of self-consciousness as a potential precursor to dancers’ use of emotional labor strategies. Self-consciousness, an important but intricate personality trait^32^, affects how one focuses attention during self-presentation^14–18^. Our research demonstrated that in general, emotional labor strategies, particularly those involving expression of genuine feelings from the heart, appeared to fall short in dancers who were inclined to direct more attention towards their outer image, suggesting that PuSC might be a potential hindrance to the brewing, generation, and modification of inner emotions. On the contrary, people who were accustomed to monitoring and evaluating their internal states (i.e., with higher PrSC) seemed to be better at engendering the expected feelings by will and hence more capable of adopting different emotional labor strategies. These results offer a preliminary understanding of how personality traits influence emotional regulation in the context of performance arts, revealing the complex dynamics of internal emotional regulation among dancers.

Thirdly, our results confirmed several preceding studies in the flow literature. We discerned positive impacts of DA and ENFE on flow experience, while SA had no such effects. This observation is in line with a previous study that directly associated emotional labor strategies with flow^59^, as well as two other studies that approached this topic indirectly^57,58^. Additionally, our insights that PrSC fosters flow experience, while PuSC fails to do so, are congruent with earlier empirical outcomes^61–64^. In light of the limited availability of relevant prior research, our study might stand as a substantial contribution to reinforcing these findings.

Lastly, thanks to the unique opportunity brought by online dance classes due to COVID-19, we were allowed to pioneer the examination of dancers' emotional regulation and expression while they danced solo. The differences between this environment and traditional performances on stage under observation could introduce a novel dimension to the dance research literature.

Recognizing the exploratory nature of our study, we advise caution in interpreting and generalizing our findings. The dynamics of emotional labor in the realm of performance arts are intricate, and what our observations provide is merely an preliminary comprehension. Future research, ideally with more varied and representative samples, is crucial to further validate our findings and to arrive at more robust conclusions.

### Practical implications

While the findings warrant cautious interpretation, this research still offers tentative practical implications. Firstly, in the realm of dance education, our study underscores the importance of focusing more on the inner self, rather than persistently responding to the evaluations of spectators, as a means to alleviate tension and bolster a sense of control on stage. Moreover, delving into the gestation and creation of authentic emotions through the Stanislavski acting method is highlighted as a more conducive and sustainable approach, especially for the well-being of dancers. Secondly, our study reinforces the scientific basis for viewing dance as a form of emotional regulation training to combat stress^78^, suggesting that a deeper immersion in the role of the dance performance is likely to yield more favorable mental health outcomes. Lastly, our research points to online dance training as a viable alternative to traditional in-person classes, particularly under the constraints of a pandemic or various other accessibility issues, given the observed similarities in dancers’ emotional regulation across both settings. Nonetheless, as these insights are preliminary and await further validation, practitioners are advised to incorporate them with discretion.

### Limitations and future research direction

Nevertheless, our study inevitably had some limitations that should be addressed by future research. The first one related to our inability to recruit more dancers as participants. Though the sample size was passable compared to other dance research^47^ given the scarcity of such participants, future replication is needed to assess the studied variables and relationships employing a more extensive and diverse sample of dancers. A larger sample would provide more statistical power, possibly testifying some of the hypotheses that we were unable to support in the current study.

The second one related to the cross-sectional nature of the data collected at a single time point. This prevents determining directionality and causal relationships between the variables. Though we hypothesized and tested a model implying certain causal sequences, the true directionality cannot be established without longitudinal or experimental data. For instance, flow experience could also plausibly influence dancers’ emotional labor strategies. Our mediation analyses should therefore be considered exploratory rather than confirmatory. Future research using longitudinal or experimental designs is essential to ascertain the causal mechanisms underlying the associations found.

Third, as we implemented a between-subject design to examine emotional labor strategies with and without being watched by spectators, there might be confounding factors uncontrolled between the two samples affecting the results, even if the dancing skills of the dancers were deemed on the same level by experts. Given that existing studies on online dancing during COVID-19 are really rare^79,80^, there is still much room for future research to continue to look into emotional labor strategies under such a unique condition of dancing in contrast to that on the stage. Though the closure of schools as a result of the pandemic has ended in China, dance students might practice dancing alone in front of a mirror or camera at ordinary times, which is also an important dance scene worthy of study.

Fourth, as this was the first study on dancers’ emotional labor strategies, merely using quantitative analyses was rough and unable to unveil the full picture, especially in terms of the distinctions between dancers’ use of emotional labor strategies and other professionals’ emotional labor strategy adoption. One important distinction was that normally, employees in service industries are asked to suppress negative emotions and to activate and express positive emotions; however, dancers are more often than not required to experience and deliver negative emotions according to the performance settings. This vast divergence might fundamentally alter the nature of emotional labor strategies and their effects on vocational well-being. Therefore, it is critical for future studies to explore the valence of expected emotions that the dancers are trying to labor. And the qualitative approach can be a more detailed exploratory attempt on this issue.

Finally, using flow alone as the outcome variable could not represent the overall performance of dancers. For one, as flow is only a subjective experience inside, dancers high in the flow state does not necessarily dance better as evaluated by the spectators or dance experts. This might account for the inconsistency between the present study and previous ones which found that PuSC was more beneficial for skillful performance under public pressure^28,29^. Thus, future studies adopting objective indicators of dancers’ performance are needed. For another, common negative indicators of mental health such as burnout, anxiety, and depression will also be needed to form and present a more complete picture of dancers’ well-being under emotional labor^81^.

## Conclusions

In this exploratory study, we ventured into the relatively unexplored domain of dancers’ emotional labor strategies as well as their antecedents in terms of self-consciousness and their outcomes in the form of flow experience. The preliminary findings primarily derived from a theoretical approach were summed as follows.

1. The dancers in our study were found to adopt a comprehensive use of the three strategies: surface acting, deep acting, and expression of naturally felt emotions, among which, deep acting was the most frequent.
2. In the condition of dancing on the stage under observation, our data suggested that expression of naturally felt emotions mediated the positive effect of private self-consciousness and the negative effect of public self-consciousness on flow experience, while surface and deep acting did not appear to function as a mediator in this context.
3. In scenarios where dancers performed without an audience, our data revealed that both deep acting and expression of naturally felt emotions mediated the positive impact of private self-consciousness on flow state, whereas public self-consciousness seemed disfunctional.

Further research, preferably with larger samples and more diverse contexts, is essential to validate and extend these initial observations.

## Data Availability

The data presented in this study are available upon request from the corresponding author because of privacy and ethical restrictions.
